# Applications of End-Tidal Carbon Dioxide (ETCO2) Monitoring in Emergency Department; a Narrative Review

**Published:** 2018-01-15

**Authors:** Hamed Aminiahidashti, Sajad Shafiee, Alieh Zamani Kiasari, Mohammad Sazgar

**Affiliations:** 1 Department of Emergency Medicine, Mazandaran University of Medical Sciences, Sari, Iran.; 2 Department of Neurosurgery, Mazandaran University of Medical Sciences, Sari, Iran.; 3 Departments of Anesthesiology and Critical Care, Mazandaran University of Medical Sciences, Sari, Iran.

**Keywords:** Capnography, Emergency service, hospital, exhalation, carbon dioxide, monitoring, physiologic

## Abstract

Capnograph is an indispensable tool for monitoring metabolic and respiratory function. In this study, the aim was to review the applications of end-tidal carbon dioxide (ETCO2) monitoring in emergency department, multiple databases were comprehensively searched with combination of following keywords: “ETCO2”, “emergency department monitoring”, and “critical monitoring” in PubMed, Google Scholar, Scopus, Index Copernicus, EBSCO and Cochrane Database.

## 1. Introduction:

 Capnometry, measuring the concentration of carbon dioxide (CO2) in the atmosphere, was used for the first time during World War II as a tool for monitoring the internal environment (1). It was used in medicine for the first time in 1950 to measure the amount of CO2 exhaled during anesthesia. However, it was not used in practice until the early 1980s and with development of smaller machines, capnometry officially entered the anesthesia field (2, 3). 

There are two types of capnograph, “side stream” and “mainstream” (4) . In the “mainstream” technique, sampling window is in the ventilator circuit and measures CO2, while in the “side stream”, the gas analyzer is located out of the ventilator circuit. In both types, gas analyzer uses infrared radiation, mass or Raman spectra and a photo acoustic spectra technology (1, 4). Flow measurement equipment is used in volumetric capnograph.   

Colorimetric CO2 detector is an example of mainstream form. These devices have a pH sensitive indicator, which changes color in inspiration and expiration. These color changes are in response to CO2 concentration changes. In the presence of a small amount of CO2, the device has a base color, which changes gradually with increase in CO2 concentration (5). 

**Figure 1:**
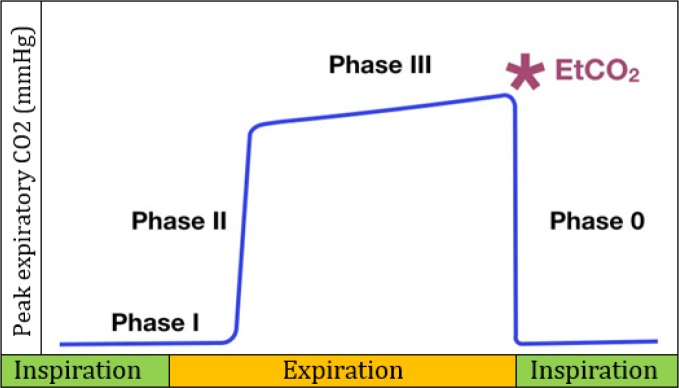
Diagram of a normal capnogram that includes the inspiratory and expiratory phase.

A normal capnograph (Figure 1) has a square-wave pattern, which begins in inspiratory phase (peak expiratory CO2 (PECO2) = 0 mmHg) and will continue until the expiratory phase (6).

***Phase 0 (inspiratory phase):*** Happens suddenly with an inspiration.

The expiration phase includes three-phases: 

***Phase I (latency phase):*** Beginning of expiration, represents anatomical dead space of the respiratory tract and is not discernible from the inspiratory phase before it (PECO2 = 0 mmHg), 

***Phase II:*** A very rapid increase in PECO2, which represents exhalation of mixed air. 

***Phase III (Plateau phase):*** Reflects the alveolar expiratory flow (a small increase in PECO2), which happens the peak at the end of tidal expiration (ETCO2). In this phase PECO2 is close to alveolar carbon dioxide tension (P_A_CO2).

Emergency physicians are always looking for a non-invasive, reliable instrument to detect life-threatening conditions in patients. One of the methods that have been suggested recently in the emergency department is capnography or ETCO2 monitoring. This study aimed to review the applications of ETCO2 monitoring in emergency department.

## 2. Evidence Acquisition

A review of the literature was performed in November 2017 to find all previously published articles that included ETCO2 application in the emergency department. The review was done in multiple electronic databases (PubMed, Google Scholar, Scopus, Index Copernicus, EBSCO and Cochrane Database) using key words including ETCO2, emergency department monitoring, and critical monitoring, searching for articles published between 1966 and 2017. In addition, all references cited in these studies were searched for the keywords. All clinical trials, case reports, case series and meta-analyses were reviewed regarding their content. In the initial search, 386 articles were found and 65 articles were eligible to be included in this review

## 3. ETCO2 Applications

**3.1. Cardio Pulmonary Resuscitation (CPR)**

ETCO2 concentration is a reliable index of effective heart compression during CPR, which is associated with cardiac output (7, 8). The first sign of the return of spontaneous circulation (ROSC) during CPR is increase in ETCO2, therefore monitoring of ETCO2 provides very useful information to guide treatment during CPR (8-10). ETCO2 is a reliable indicator with a high prognostic value in determining the CPR outcome (11, 12) . ​​Studies have shown that in patients who had ETCO2 of 10 mmHg or less, cardiac arrest was associated with death (13, 14). After 20 minutes of CPR, death occurs if ETCO2 is consistently below 10 mmHg, with 100% sensitivity and specificity (15). ETco2 is more sensitive than cerebral oxygen saturations (rSO_2_) in ROSC prediction (16).

**3.2. Airway assessment**

Confirmation of endotracheal intubation is vital in airway management in the emergency department, while there is no definitive diagnostic tool to verify correct intubation in emergency rooms (17). Recently, capnography was used as the gold standard for confirming the correct location of the endotracheal tube (18, 19). Colorimetric ETCO2 is a safe, reliable, simple and portable tool to determine the proper placement of endotracheal tube in patients with stable hemodynamic and it is very useful when a capnograph is not available (20) . However, when patients have a bag or mask ventilation or consume carbonated beverages or antacids it can cause a false positive result, yet it usually indicates the true result after 6 breaths (21). The use of sodium bicarbonate leads to a higher level of ETCO2 for 5 to 10 minutes (22). During a cardiac arrest, which leads to decrease in tissue-pulmonary CO2 transportation, capnography can show a correct intubation as a wrong one (false negative)(23).

**3.3. Procedural sedation and analgesia**

Capnography is an effective method to diagnose early respiratory depression and airway disorders, especially during sedation, leading to a reduction in serious complications (23, 24). Capnography provided more safety in monitoring patients during sedation. Oxygen prescription does not have an effect on respiratory function parameters evaluated by capnography (25). It shows impaired airway function sooner than any other device, 5 to 240 seconds earlier than pulse oximetry (26, 27). Capnography is more sensitive than clinical evaluation in diagnosis of respiratory dysfunction, for instance, in many cases where apnea was experienced during sedation, doctors at the bedside did not recognize the apnea but capnography could identify it (28).

**3.4. Pulmonary disease**

**3.4.1. Obstructive pulmonary disease**

In obstructive airway diseases, hypoventilation can cause shortness of breath and hypercarbia (29). There is a relationship between ETCO2 and partial arterial carbon dioxide (PaCO2) in patients with acute asthma in the emergency department (30, 31). Capnography is dynamic monitoring of patients with acute respiratory distress conditions such as asthma, chronic obstructive pulmonary disease (COPD), bronchiolitis, and heart failure (32). Bronchospasm is associated with a prolonged expiratory phase (E1, E2, E3) in capnogram (Figure 1) in patients with obstructive diseases such as COPD (32, 33). Changes in ETCO2 and expiratory phase slope correlated with (E1, E2, E3) forced expiratory volume in 1 second (FEV1) and Peak expiratory flow rate (PEFR) (32, 34). ETCO2 is an indispensable tool in assessing the severity of obstructive respiratory disease in the emergency department. ETCO2 is higher in patients with COPD exacerbation who are admitted to the hospital compared to those who are discharged from the emergency department (35).

**3.4.2. Pulmonary embolism**

In thromboembolism, ETCO2 is significantly lower than normal due to the reduction of pulmonary perfusion and increased alveolar dead space that reduces the amount of CO2 exhaled from the lungs, so venous carbon dioxide pressure (PvCO2) increases and all of these changes lead to an increase in arterial CO2-ETCO2 gradient (36). This helps in correctly diagnosing pulmonary embolism, especially silent pulmonary embolism (37). Volumetric capnography is used for monitoring of thrombolysis in large pulmonary embolism (38). The average value of ETCO2 and decrease in PCO2 / PO2 pressure for 30 seconds correlates with clinical probability or rule out of pulmonary embolism (39).

**3.5. Heart failure**

Rapid differentiation of heart failure as the cause of dyspnea from other respiratory causes, is very important for choosing an appropriate therapy (40). Sometimes distinguishing COPD / asthma exacerbation and acute heart failure is very difficult, especially when both exist together, and treatment decisions in this situation are very complex (41). ETCO2 in patients with cardiac causes is markedly different from patients with respiratory distress due to obstructive causes. ETC02 level > 37 mmHg was not observed in any patient with heart failure, although ETC02 level > 37 mmHg has a slight sensitivity for diagnosis of COPD / asthma (42, 43). ETCO2 level during cardiopulmonary exercise testing in patients with heart failure has high prognostic value for cardiac events (44, 45). N-Terminal Pro- brain Natriuretic Peptide on the side of quantitative capnography is very useful in early diagnosis and treatment of patients with acute dyspnea (respiratory or cardiac causes) in emergency departments. The widespread use of quantitative capnography can be beneficial in everyday work for emergency physicians (46).

**3.6. Shock**

Hypotensive shock is a clinical feature for many diseases and is related to high mortality rate in emergency departments. Emergency physicians continuously strive to find new ways to diagnose early-stage shock to start treatment as soon as possible (47). Capnography is considered as a simple and non-invasive method to detect and estimate shock intensity in the early stage (48, 49). ETCO2 is known to be decreased in volume-related hypotensive states (50). ETCO2 has a correlation with blood pressure, serum lactate and base excess. In early-stage shock that is linked to reduced cardiac output, the amount of ETCO2 significantly decreases. This is due to decreased blood flow in the pulmonary artery during the cardiac output reduction, which disrupts ventilation perfusion ratio. With increase in shunt ETCO2 level decreases, while P_a_CO2 does not change (51, 52). With decrease in blood pressure, ETCO2 drops and P_a_CO2-ETCO2 gradient increases (53, 54). There is a correlation between the amount of dehydration and the amount of sodium bicarbonate and ETCO2, and ETCO2 can be used as a simple and non-invasive indicator for determination of dehydration (55).

**3.7. Metabolic disorder**

Carbon dioxide (CO2) is one of the final products of metabolism and is transferred to lungs through the blood circulation and transmitted through respiratory system, so exhaling CO2 reflects the body’s metabolic status (56, 57). ETCO2 is a fast, inexpensive and non-invasive indicator to estimate the amount of HCO3- bicarbonate and PaCO2 in emergency and critical situations (58). Due to the direct connection between ETCO2 and HCO3, ETCO2 is a predictor of metabolic acidosis and mortality, so capnograph as a screening tool for metabolic acidosis is very useful in the emergency department (59) . ETCO2 can be recommended as a noninvasive method for determination of metabolic acidosis and can be used to detect early metabolic acidosis in patients with spontaneous breathing, however, ABG should be used as the gold standard for diagnosis and management of treatment (60). 

**3.7.1. Diabetic keto acidosis (DKA)**

Patients with diabetes mellitus are at increased risk of major and disabling complications, one of the most important of which is DKA (61). The Direct linear relationship between ETCO2 and HCO3 is useful in prediction of acidosis. It was shown that there is no DKA diagnosis when ETCO2 ˃ 36, and there is DKA diagnosis when ETCO2 ≤ 29. ETCO2 30 to 35 is considered as the cut –point, so it is clinically useful in diagnosis of acidosis (62, 63). In addition, a low P_a_CO2 level is correlated with increased risk of cerebral edema in children with DKA (64). Thus, according to the relationship between ETCO2 and P_a_CO2, capnography can be used to identify individuals with high risk of cerebral edema (62). When the patient's glucose is above 550 mg/dl, ETCO2 is a useful tool to rule out the DKA (65).

**3.7.2. Gastroenteritis**

Among children with diarrhea and vomiting, ETCO2 is independently correlated with serum HCO3 concentration. This is a non-invasive index for measuring the severity of acidosis in patients with gastroenteritis (66). ETCO2 can be used to estimate HCO3 in many emergency situations(58).

**3.8. Trauma**

End-tidal carbon dioxide cannot be used to rule out severe injury in patients meeting the criteria for trauma care. ETCO_2_ ≤30 mmHg may be associated with increased risk of traumatic severe injury (67). 

There is a reverse relationship between pre-hospital ETCO2 and traumatic mortality rates, so ETCO2 can be used to improve triage and also helps the emergency medical service staff in planning for the transfer of patients to the appropriate trauma center (68).

Low ETCO2 has a strong association with shock in patients with trauma and suggests the severity of the patient's condition in the first 6 hours of admission (69).

## 4. Conclusion:

ETCO2 is used in the emergency department as an indicator for measurement in many clinical situations. Capnography is a non-invasive and accurate method to measure ETCO2 and can help emergency physicians in some critical situations. Although this is not used in many emergency situations and it is not used routinely in the emergency department, its application is increasing in many emergency situations, such as patients undergoing mechanical ventilation, procedural sedation and analgesia, pulmonary disease, heat failure, shock, metabolic disorder and trauma. This means that capnography must be considered as an essential tool in emergency department, however, more researches are needed to evaluate its application in specific clinical conditions and diseases. 
